# Management of Non-syndromic Multiple Impacted Teeth with Dentigerous Cysts: A Case Report

**DOI:** 10.7759/cureus.3323

**Published:** 2018-09-18

**Authors:** Kishore Moturi, Vini Kaila

**Affiliations:** 1 Oral and Maxillofacial Surgery, Vishnu Dental College, Bhimavaram, IND

**Keywords:** non syndromic - multiple impacted teeth, impactions, multiple impacted teeth, dentigerous cysts, posterior iliac graft

## Abstract

Impacted teeth may not only interfere with function, but also can act as a source of many pathological lesions such as odontogenic cysts and tumors. One of the most prevalent types of odontogenic cysts associated with erupted, developing or impacted tooth is dentigerous cyst. Multiple impacted teeth with dentigerous cysts in both the maxillary and mandibular arches without the association of any syndromes is a very rare occurrence. In the present article, we report such a non-syndrome case of bilateral multiple impacted teeth in both maxilla and mandible with dentigerous cysts treated with enucleation and ridge augmentation with autogenous bone graft harvested from posterior iliac region. Further, dental rehabilitation was carried out with dentures.

## Introduction

Teeth form the functional and aesthetic unit of the mouth. Any disorder in the formation or eruption of these functional units results in many problems to the human body. Eruption is the physiologic process that moves a tooth from its crypt position through the alveolar process into the oral cavity to its final position of occlusion. It is a dynamic process which consists of root completion, formation of the periodontium and maintenance of a functional occlusion [1]. Teeth that are prevented from erupting by some physical barrier are called impacted teeth. This may occur due to various etiological factors including lack of space due to crowding of dental arches or premature loss of deciduous teeth. Rotation or other positional deviations of tooth buds may result in teeth that are “aimed” in wrong direction, leading to impaction [2].

## Case presentation

**Figure 1:**
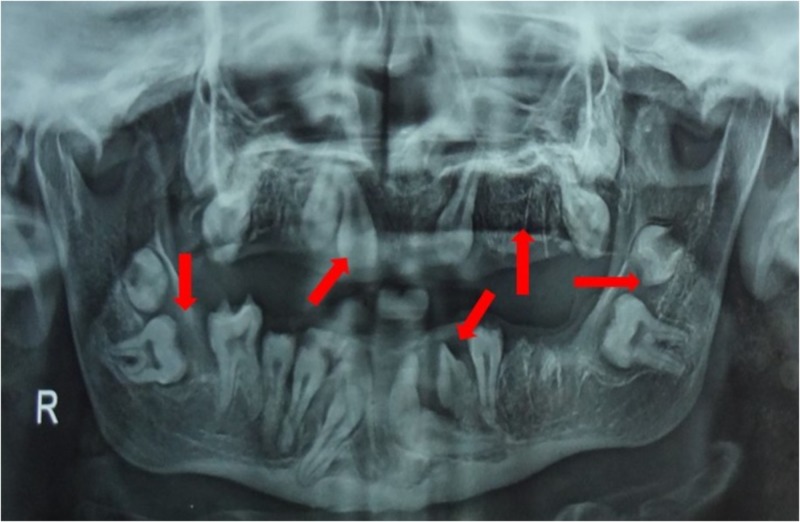
Pre-operative orthopantomogram. Preoperative radiograph demonstrating multiple impacted teeth in both maxilla and mandible, extraction sockets in the maxilla bilaterally and in the mandible on left side.

A 19-year-old female patient reported to the Department of Oral and Maxillofacial Surgery, Vishnu Dental College, Bhimavaram with multiple impacted teeth. She gave a previous history of extractions of teeth. Her medical history revealed two episodes of epileptic seizures in the past. The patient was not under medication for any known disease at time of presentation. She was poorly built with a short stature. Extra oral examination revealed frontal bossing with medial squint of the eye. The maxilla and mandible were hypoplastic. The clinical oral examination revealed severely resorbed maxillary and mandibular arches with irregular alveolar ridge height and shallow buccal and lingual vestibules. Orthopantomogram demonstrated bilateral multiple radiolucent lesions associated with impacted teeth and healing sockets in both the arches (Figure 1).

Computed tomography revealed abnormal bone morphology and calvarial suture pattern. The maxillary bone appeared highly dense with atypical trabecular pattern. Her biochemical profile was normal except for increased alkaline phosphatase value. Karyotyping was performed to rule out the association of any syndrome and the result was negative.

Pathological examination

Surgical extraction of an impacted tooth and incisional biopsy of lesion and bone was done in the region of 14 and 15. The pathological analysis revealed the lesion as dentigerous cyst with no abnormality in bone and tooth.

Treatment

**Figure 2:**
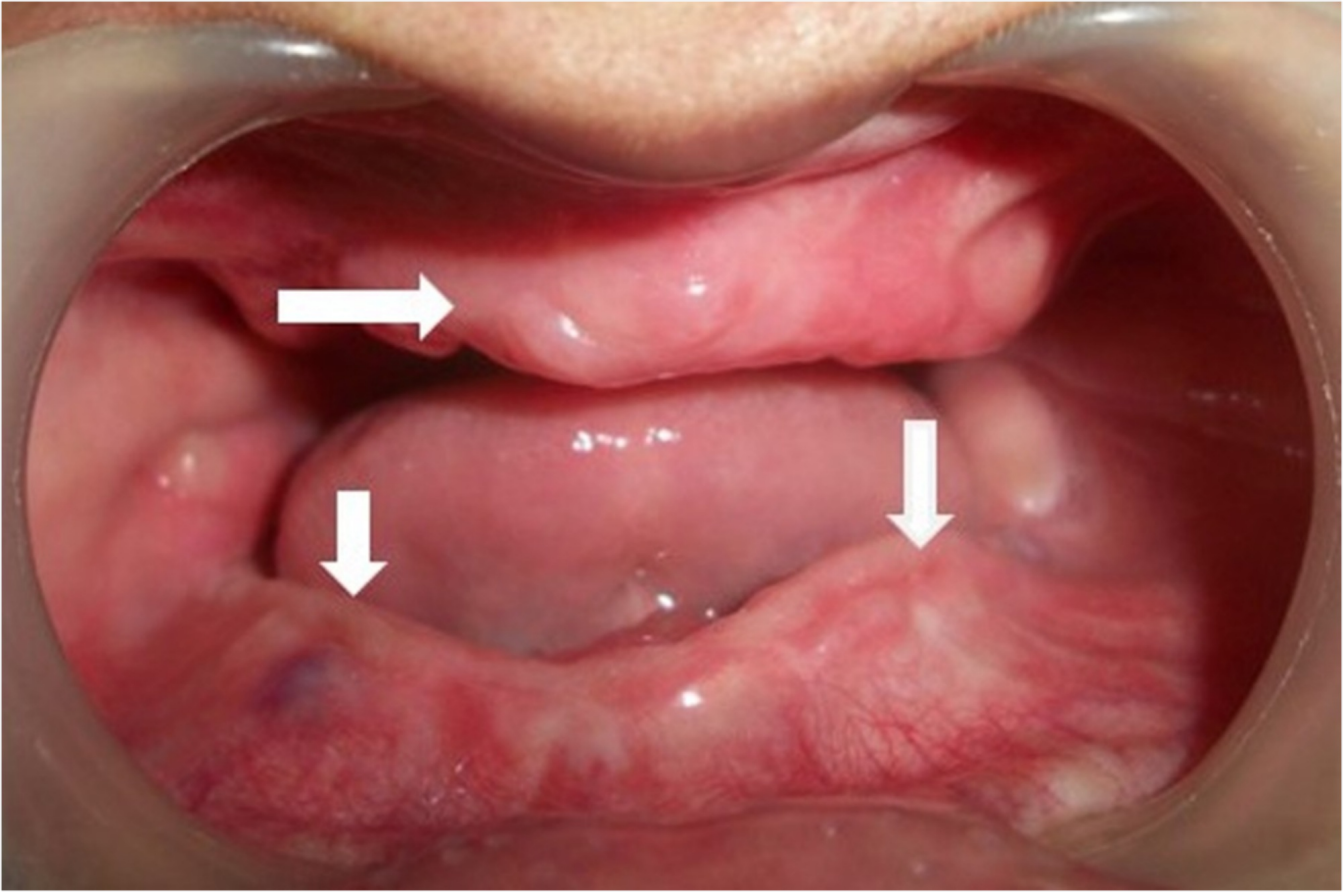
Post-operative intra oral view. Autogenous bone graft augmented ridges.

Surgical extraction of all the impacted teeth and enucleation of the associated cysts was done under general anaesthesia. Ridge augmentation was performed with the autogenous bone graft harvested from posterior iliac crest region (Figure 2).

**Figure 3:**
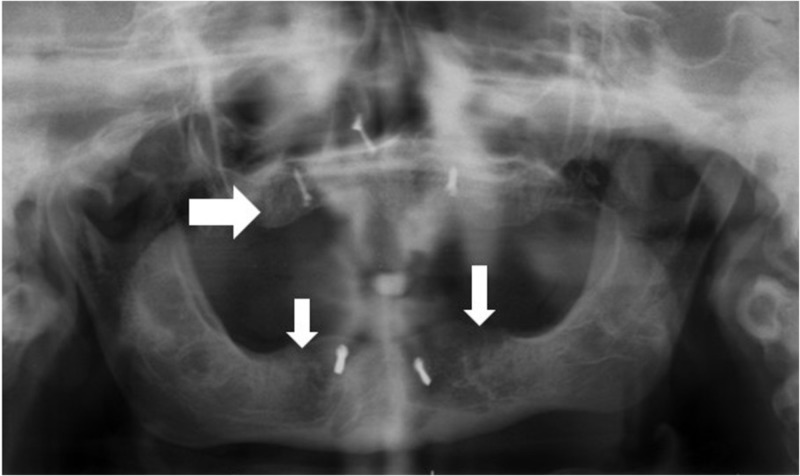
Post-operative radiograph.

Post-operative orthopantomogram shows good healing of the grafted bone without recurrence of the cyst (Figure 3).

**Figure 4:**
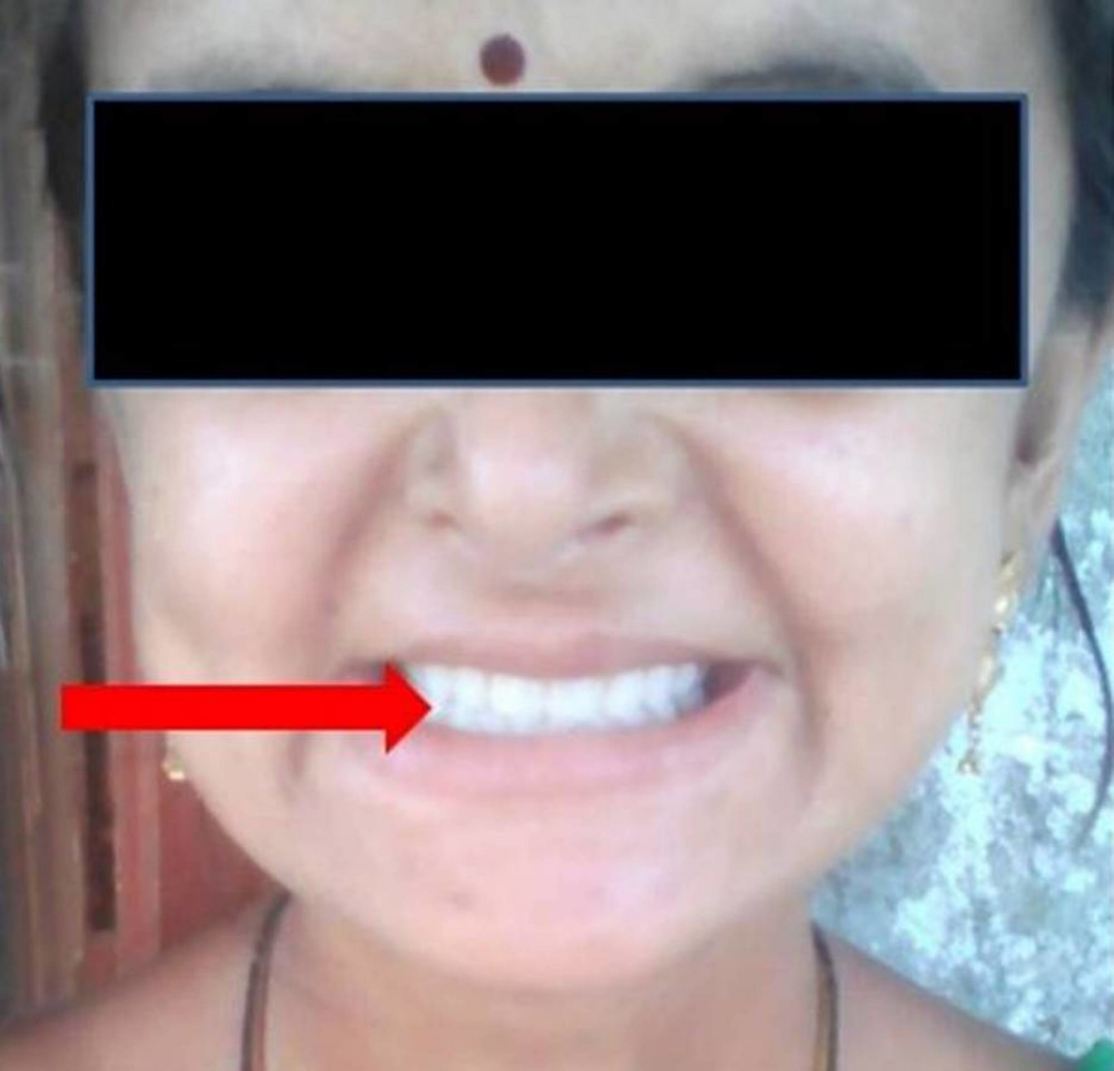
Post-operative photograph of the patient with the interim dentures.

Interim complete dentures were fabricated in the immediate postoperative period for aesthetic purpose (Figure 4).

The patient was further planned for full mouth rehabilitation with implant-based prosthesis at a later stage.

## Discussion

Eruption is the axial movement of a tooth from its position in the bone to its final functional occlusion in the oral cavity [1]. However, eruption is often used to indicate the moment of emergence of the tooth into the oral cavity. The normal eruption of deciduous and permanent teeth into the oral cavity occurs over a broad chronologic age range. It can also be influenced by racial, ethnic, sexual and individual factors and these are usually considered in determining the standards of normal eruption [3]. There are numerous eruption regulating molecules having similar and overlapping functions, which ensures that even the absence of a single factor does not interrupt the event of eruption. EGF, EGF-R, CSF-1, CSF-1R, IL-1, IL-1R, c-Fos, NFB, MCP-1, TGF-b1, PTHrP, Cbfa-1, OPG, RANK/RANK L are the major tooth eruption molecules. Majority of the eruption molecules reside in the dental follicle with few in the Stellate reticulum [4]. But, defect in some genes may be responsible for abnormalities in the eruption. Significant deviations from accepted norms of eruption sequence are often observed in clinical practice. Premature eruption has been noted but delayed tooth eruption is the most commonly encountered [2]. In normal eruption scenario, permanent teeth erupt eventually and replace their primary predecessors. However, some teeth fail to erupt. Most of these unerupted teeth are deviated or angulated aberrantly and eventually lose their potential to erupt and are referred to as impacted teeth. Epidemiological studies have reported dental impactions to affect 25 to 50% of human population [3]. Impaction of teeth can result from biomechanical impediments, previous dento-alveolar trauma, crowding and malpositioning of adjacent teeth, thickened mucosal and osseous tissues, insufficient maxillofacial skeletal development, eruption disturbances, indirect effects of cysts or neoplasms [5]. Impaction of a single tooth is a commonly observed clinical finding but impaction of multiple teeth is uncommon. Multiple impacted teeth with no obvious etiology is a rare dental anomaly. In literature, few reports are related to multiple impacted teeth with no known etiology [6].

Multiple impacted teeth by itself is often associated with multiple syndromes such as Cleidocranial dysplasia, Gardners syndrome, Gorlin-Sedano syndrome, Yunis-Varon syndrome, Mucopolysaccharides [4]. It is also common in endocrine disorders such as hypothyroidism, hypopituitarism, hypoparathyroidism. Metabolic disorders like Vitamin-D deficiency are also associated with impacted teeth.

Multiple bilaterally impacted teeth in both maxillary and mandibular arches is a rare entity. When such multiple impacted teeth are present, association with any pathology such as dentigerous cysts, syndromes, metabolic and hormonal disorders in these patients should be ruled out. Dentigerous cysts are the most common true cysts of the jaws accounting for approximately 24% of all true cysts [7]. The cyst involves the odontogenic epithelia of impacted permanent teeth, supernumerary teeth and rarely deciduous teeth.

When there are bilateral multiple impacted teeth with associated cysts in both the arches, the orthopantomograph may not be of great aid. Computed tomographic scans provide superior detail of the hard tissue, allowing for the visualization of the size and extent of the lesion with determination of orbital or nasal invasion or involvement.

Management of multiple impacted teeth associated with dentigerous cysts involves a complex multidisciplinary approach. Age of the patient, position of the teeth and number of impacted teeth associated with dentigerous cysts and any concomitant metabolic, genetic and syndromic abnormalities have to be considered during the treatment planning. Guided eruption of many teeth with the help of coordinated multidisciplinary management is needed for patients with multiple impactions [8]. Damage to the involved permanent teeth can be prevented by enucleation of smaller lesions. Larger lesions may be decompressed and marsupialized to relieve the pressure within the cysts followed by enucleation when the size has decreased which is also known as Waldron's procedure [9,10].

Following complete removal, dentigerous cysts are known to recur very rarely [11]. This is related to the exhausted nature of the reduced enamel epithelium, which has differentiated and formed tooth crown enamel before becoming a cyst [12]. The present case had multiple small and large cysts involving single and multiple teeth both in maxillary and mandibular arches. Orthodontic associated eruption was ruled out as the patient was not willing for prolonged treatment option. Extractions, enucleation and reconstruction of the alveolar defects with autogenous bone graft was planned. As the size of the defect was large, posterior iliac crest was selected as the donor site. For more than four decades, the iliac crest has been the favourable donor site to harvest bone for augmentation in orthopedic, neurosurgery and maxillofacial surgery. In oral and maxillofacial surgery, the main indications are secondary and tertiary osteoplasty in patients with cleft lip and palate, augmentation of bony defects after operations for tumors or large cysts, and augmentation in preprosthetic surgery for severe cases of atrophy of the alveolar crest from early loss of teeth or the aging process. The main advantages of the iliac crest are its easy accessibility, the possibility of harvesting large amounts of bone, and the ability to close the wound primarily. The anterior iliac crest is more accessible than the posterior iliac crest as a donor region and bi-cortical grafts can also be harvested. The bi-cortical bone layers between the internal and external cortices below the internal anterior iliac crest are as thin as a blade, often making it difficult to obtain a sufficient bone volume. As the bi-cortical width of the bone layer beneath the posterior iliac crest does not shrink, this region always offers enough cortical and cancellous bone for augmentation even in cases of extreme alveolar atrophy or for the reconstruction of large bony defects [13]. The average volume of bone graft harvested from anterior and posterior sites has been reported to be 13 cm^3^ and 30 cm^3^ respectively [14]. Nkenke et al. compared the morbidity of harvesting of bone grafts from anterior iliac crest with that of posterior iliac crest for pre-prosthetic augmentation procedures and reported that posterior iliac crest harvesting has less morbidity and fewer complications compared to the anterior crest technique [15]. Though the surgical technique for bone harvesting from anterior and posterior iliac crests is fairly simple, the posterior iliac crest harvesting requires the patient to be turned around during the operation which requires two teams and also extended operating time.

## Conclusions

Multiple impacted teeth that are asymptomatic can be evaluated periodically with radiographs. Apart from various treatment modalities available for treatment of dentigerous cysts, such as decompression and guided tooth eruption, enucleation with ridge augmentation using an iliac graft as reported in the present case, can be considered in cases with multiple impacted teeth and associated dentigerous cysts depending on the cyst size and location. Further, dental rehabilitation with implant supported dentures help improve the quality of life of these patients.
